# Supplementary material for: Prevalence of psychiatric disorders in Norwegian 10-14-year-olds: Results from a cross-sectional study
Source: PLoS One. 2021 Mar 19;16(3):e0248864. doi: 10.1371/journal.pone.0248864 (PMC7978367; doi:10.1371/journal.pone.0248864)
Supplement: S1 Table — (DOCX) [file pone.0248864.s001.docx]

**S1 Table. Prevalence of DSM-IV disorders among 10-14-year-old participants in the Bergen Child Study.**

|  | Unweighted^1^ | | Weighted to population^2^ | |
| --- | --- | --- | --- | --- |
|  | % (n) | 95% CI | % | 95% CI |
| Any psychiatric disorder | 5.87 (80) | 4.73-7.25 | 6.93 | 5.06-9.41 |
| Any anxiety disorder | 2.86 (39) | 2.10-3.89 | 3.84 | 2.50-5.85 |
| Separation anxiety | 0.22 (3) | 0.07-0.68 | 0.73 | 0.20-2.63 |
| Specific Phobia | 1.03 (14) | 0.61-1.73 | 0.96 | 0.52-1.78 |
| Social phobia | 0.29 (4) | 0.11-0.78 | 0.22 | 0.07-0.70 |
| OCD | 0.22 (3) | 0.07-0.68 | 0.41 | 0.13-1.31 |
| Generalised anxiety | 0.07 (1) | 0.01-0.52 | 0.03 | 0.00-0.24 |
| Other anxiety | 1.03 (14) | 0.61-1.73 | 1.49 | 0.71-3.08 |
| Any depressive disorder | 0.44 (6) | 0.20-0.98 | 0.64 | 0.16-2.58 |
| Major depression | 0.29 (4) | 0.11-0.78 | 0.59 | 0.13-2.65 |
| Other depression | 0.15 (2) | 0.04-0.59 | 0.04 | 0.01-0.18 |
| Any ADHD | 1.17 (16) | 0.72-1.91 | 1.54 | 0.76-3.10 |
| ADHD combined | 0.59 (8) | 0.29-1.17 | 0.92 | 0.32-2.65 |
| ADHD inattentive | 0.29 (4) | 0.11-0.78 | 0.36 | 0.12-1.03 |
| ADHD hyp-imp | 0.07 (1) | 0.01-0.52 | 0.10 | 0.01-0.68 |
| Other hyperactivity | 0.22 (3) | 0.07-0.68 | 0.17 | 0.04-0.67 |
| Any disruptive disorder | 1.32 (18) | 0.83-2.09 | 1.62 | 0.82-3.18 |
| Oppositional defiant | 0.44 (6) | 0.20-0.98 | 0.69 | 0.19-2.56 |
| Conduct disorder | 0.29 (4) | 0.11-0.78 | 0.24 | 0.07-0.81 |
| Other disruptive | 0.59 (8) | 0.29-1.17 | 0.69 | 0.31-1.55 |
| Any other psychiatric disorder | 1.39 (19) | 0.89-2.17 | 1.09 | 0.61-1.95 |
| PDD/Autism | 1.17 (16) | 0.72-1.91 | 0.90 | 0.47-1.73 |
| Tic disorder | 0.22 (3) | 0.01-0.07 | 0.19 | 0.05-0.70 |
| ^1^DAWBA participants only. | | | | |
| ^2^Weighted to population margins for education levels in the population | | | | |

^1^DAWBA participants only.

^2^Weighted to population margins for education levels in the population.

Note. DSM-IV diagnoses not listed in the table above were not diagnosed among participants in the current study. The dash (“-”) indicates that the diagnosis was not made for that gender.
