# Supplementary material for: Prevalence of psychiatric disorders in Norwegian 10-14-year-olds: Results from a cross-sectional study
Source: PLoS One. 2021 Mar 19;16(3):e0248864. doi: 10.1371/journal.pone.0248864 (PMC7978367; doi:10.1371/journal.pone.0248864)
Supplement: S2 Table — (DOCX) [file pone.0248864.s002.docx]

**S2 Table. Prevalence of DSM-IV disorders among 10-14-year-old participants in the Bergen Child Study, by gender.**

|  | **Unweighted^1^** | | | | **Weighted to population^2^** | | | |
| --- | --- | --- | --- | --- | --- | --- | --- | --- |
|  | Girls | | Boys | | Girls | | Boys | |
|  | % | 95% CI | % | 95% CI | % | 95% CI | % | 95% CI |
| Any psychiatric disorder | 4.64 | 3.32-6.46 | 7.20 | 5.45-9.46 | 7.52 | 4.68-11.86 | 6.27 | 4.42-8.83 |
| Any anxiety disorder | 2.81 | 1.82-4.32 | 2.91 | 1.86-4.52 | 4.37 | 2.35-8.00 | 3.25 | 1.93-5.40 |
| Separation anxiety | 0.14 | 0.02-1.00 | 0.31 | 0.08-1.22 | 0.86 | 0.13-5.66 | 0.58 | 0.14-2.34 |
| Specific Phobia | 0.98 | 0.47-2.05 | 1.07 | 0.51-2.24 | 1.14 | 0.49-2.62 | 0.76 | 0.31-1.87 |
| Social phobia | 0.42 | 0.14-1.30 | 0.15 | 0.02-1.08 | 0.31 | 0.08-1.27 | 0.12 | 0.02-0.83 |
| OCD | - | - | 0.46 | 0.15-1.42 | - | - | 0.86 | 0.27-2.74 |
| Generalised anxiety | - | - | 0.15 | 0.02-1.08 | - | - | 0.07 | 0.01-0.51 |
| Other anxiety | 1.27 | 0.66-2.42 | 0.77 | 0.32-1.83 | 2.06 | 0.80-5.19 | 0.85 | 0.33-2.20 |
| Any depressive disorder | 0.56 | 0.21-1.49 | 0.31 | 0.08-1.22 | 1.13 | 0.25-4.96 | 0.09 | 0.02-0.38 |
| Major depression | 0.42 | 0.14-1.30 | 0.15 | 0.02-1.08 | 1.09 | 0.23-5.04 | 0.05 | 0.01-0.33 |
| Other depression | 0.14 | 0.02-1.00 | 0.15 | 0.02-1.08 | 0.04 | 0.01-0.30 | 0.05 | 0.01-0.33 |
| Any ADHD | 0.56 | 0.21-1.49 | 1.84 | 1.04-3.21 | 1.61 | 0.50-5.06 | 1.47 | 0.73-2.91 |
| ADHD combined | 0.28 | 0.07-1.12 | 0.92 | 0.41-2.03 | 1.18 | 0.26-5.22 | 0.62 | 0.22-1.74 |
| ADHD inattentive | 0.28 | 0.07-1.12 | 0.31 | 0.08-1.22 | 0.42 | 0.11-1.67 | 0.28 | 0.05-1.48 |
| ADHD hyp-imp | - | - | 0.15 | 0.02-1.08 | - | - | 0.20 | 0.03-1.43 |
| Other hyperactivity | - | - | 0.46 | 0.15-1.42 | - | - | 0.36 | 0.09-1.41 |
| Any conduct/oppositional disorder | 0.56 | 0.21-1.49 | 2.14 | 1.27-3.59 | 1.19 | 0.28-4.91 | 2.10 | 1.11-3.95 |
| Oppositional defiant | 0.14 | 0.02-1.00 | 0.77 | 0.32-1.83 | 0.86 | 0.12-5.75 | 0.51 | 0.17-1.48 |
| Conduct disorder | 0.14 | 0.02-1.00 | 0.46 | 0.15-1.42 | 0.18 | 0.03-1.29 | 0.30 | 0.06-1.46 |
| Other disruptive | 0.28 | 0.07-1.12 | 0.92 | 0.41-2.03 | 0.15 | 0.03-0.66 | 1.30 | 0.53-3.14 |
| Any other psychiatric disorder | 0.42 | 0.14-1.30 | 2.45 | 1.50-3.97 | 0.13 | 0.04-0.44 | 2.17 | 1.16-4.00 |
| PDD/Autism | 0.42 | 0.14-1.30 | 1.99 | 1.16-3.40 | 0.13 | 0.04-0.44 | 1.77 | 0.88-3.54 |
| Tic disorder | - | - | 0.46 | 0.15-1.42 | - | - | 0.40 | 0.11-1.47 |

^1^DAWBA participants only.

^2^Weighted to population margins for education levels in the population.

Note. DSM-IV diagnoses not listed in the table above were not diagnosed among participants in the current study. The dash (“-”) indicates that the diagnosis was not made for that gender.
