# Supplementary material for: Prevalence of psychiatric disorders in Norwegian 10-14-year-olds: Results from a cross-sectional study
Source: PLoS One. 2021 Mar 19;16(3):e0248864. doi: 10.1371/journal.pone.0248864 (PMC7978367; doi:10.1371/journal.pone.0248864)
Supplement: Attachment — Submitted filename: rebuttal.docx [file pone.0248864.s003.docx]

To Editor-in-chief, PLOS ONE.

We would like to thank you for the opportunity to revise our manuscript. Below you will find our responses to the reviewer’s concerns, our responses in *italics*. We have uploaded a marked copy of our manuscript, as well as an unmarked version to the submission system, accompanying this rebuttal letter.

We would also like to thank the academic editor for extending the deadline for resubmission of our manuscript.

Yours sincerely,

Tormod Bøe, PhD

On behalf of all authors.

1. Please ensure that your manuscript meets PLOS ONE's style requirements, including those for file naming. The PLOS ONE style templates can be found at

https://journals.plos.org/plosone/s/file?id=wjVg/PLOSOne_formatting_sample_main_body.pdf and

https://journals.plos.org/plosone/s/file?id=ba62/PLOSOne_formatting_sample_title_authors_affiliations.pdf

*We have now updated the manuscript according to the PLOS ONE style requirements.*

2.Thank you for including your ethics statement:  "The study was approved by the Regional Committee for Medical Research Ethics in Western Norway (approval number 062-06) and by the National Data Inspectorate.".

Please provide additional details regarding participant consent. In the ethics statement in the Methods and online submission information, please ensure that you have specified (1) whether consent was informed and (2) what type you obtained (for instance, written or verbal, and if verbal, how it was documented and witnessed). If your study included minors, state whether you obtained consent from parents or guardians. If the need for consent was waived by the ethics committee, please include this information.

(…)

Once you have amended this/these statement(s) in the Methods section of the manuscript, please add the same text to the “Ethics Statement” field of the submission form (via “Edit Submission”).

*We have now updated the ethics statement in the manuscript, and in the “Ethics statement” field of the submission form. The revised statement now reads:*

*"The study was approved by the Regional Committee for Medical Research Ethics in Western Norway (approval number 062-06) and by the National Data Inspectorate. Participants gave written informed consent and parents and guardians consented on behalf of their children".*

3. Please amend either the title on the online submission form (via Edit Submission) or the title in the manuscript so that they are identical.

*We apologize for this error; titles are now identical in the submission form and in the manuscript.*

4. Please amend your list of authors on the manuscript to ensure that each author is linked to an affiliation. Authors’ affiliations should reflect the institution where the work was done (if authors moved subsequently, you can also list the new affiliation stating “current affiliation:….” as necessary).

*The list of author affiliations has been updated.*

5.We note that you have indicated that data from this study are available upon request. PLOS only allows data to be available upon request if there are legal or ethical restrictions on sharing data publicly. For information on unacceptable data access restrictions, please see http://journals.plos.org/plosone/s/data-availability#loc-unacceptable-data-access-restrictions.

In your revised cover letter, please address the following prompts:

a) If there are ethical or legal restrictions on sharing a de-identified data set, please explain them in detail (e.g., data contain potentially identifying or sensitive patient information) and who has imposed them (e.g., an ethics committee). Please also provide contact information for a data access committee, ethics committee, or other institutional body to which data requests may be sent.

b) If there are no restrictions, please upload the minimal anonymized data set necessary to replicate your study findings as either Supporting Information files or to a stable, public repository and provide us with the relevant URLs, DOIs, or accession numbers. Please see http://www.bmj.com/content/340/bmj.c181.long for guidelines on how to de-identify and prepare clinical data for publication. For a list of acceptable repositories, please see http://journals.plos.org/plosone/s/data-availability#loc-recommended-repositories.

We will update your Data Availability statement on your behalf to reflect the information you provide.

*We have updated the Data availability statement to detail the legal restrictions regarding sharing of the data set and also provide this information in the cover letter. The data availability statement now reads:*

*“Data cannot be shared publicly due to privacy restrictions in accordance with the ethical approval for the Bergen Child Study. Norwegian Health research legislation and the Norwegian Ethics committees require explicit consent from participants in order to transfer health research data outside of Norway. In this specific case, ethics approval is also contingent on storing the research data on secure storage facilities located in our research institution. Data are from the Norwegian Bergen Child Study, owned by NORCE Norwegian Research Centre. The authors did not have special access privileges to data from the survey.* Data are available from the *Bergen Child Study* (contact via *bib@norceresearch.no*) for researchers who meet the criteria for access to confidential data.*”*

Reviewers' comments:

Reviewer #1: In this paper "Prevalence of Psychiatric Disorders in Norwegian 10-14 Year-Olds". In this paper, The aim of this study to find prevalence of psychiatric disorders in a Norwegian population-based sample of early adolescent boys and girl. Authors have highlighted limitation of official clinical and administrative registries. Authors also reported prevalence of psychiatric disorder in same cohort between age 7-9 years old in the previous study. I have following concerns about this study.

Method section:

One of the major limitations of this study only 24% completed the full assessment (DAWDA), while this population base study - but only 1/4 completed the assessment and also who completed have more parents education. Authors already reported that about lower socioeconomic status are under-represented as limitation of administrative registry. So how this sample is more representative?

*In the authors’ opinions, the strength of the current study is the use of weighting to counter the selective participation by higher educated participants commonly seen in previous literature. By using weights, we use the information about those not participating to estimate what the prevalence would have been if all participants had completed the clinical interview.*

2nd paragraph, authors report that teacher’s data from the screening phase is not included. However, in abstract and next paragraph (DAWBA was administered through a web-based system, and parents and teachers provided information), and psychiatric assessment section: authors report teacher report was included.

*This is correct. Teacher data was not included in the screening phase (see Figure 1), but it was included in the psychiatric assessment phase. In the current study, information from the screening phase is used for weighting (parental education levels, reported by parents) and to indicate representativeness of the sample participating in the psychiatric assessment to the full sample (results described under own heading on page 5).*

What the rationale of dividing the groups in so close age range? [10.4,11.7), [11.7,12.2), [12.2,12.8), [12.8,13.4) and [13.4,14.2].

*The rationale for dividing the age groups in this way were to provide a detailed perspective on the prevalence of different disorders across this narrow age span. As discussed, (p. 6) the prevalence was highly variable depending on how age was categorized, and we therefore also report the main effects of (uncategorized) age (and its interaction with gender) in the manuscript (pp. 6-7).*

Discussion section:

Authors reports low prevalence related to methodological issues as sampling procedure and choice of diagnostic instrument. If we have same issues with this study - how it is better and adding to what we already know?

*As detailed above, the weighting procedure used in the current study is aimed at alleviating problems with sampling procedures from previous studies. Regarding the use of DAWBA, this is merely a point to consider when interpreting the results from the current study, and when comparing and contrasting this study to previous studies where different instruments may have been used.*

Authors also reports that low prevalence may be related to DAWDA time frame "current". However, ADHD is chronic psychiatric disorder and anxiety could be also pervasive in childhood ; so how it is different CAPA, DISC or CIDI. Current time frame in DAWDA is also very vague. Authors themselves highlighted the limitation of DAWBA (....only half the number of DSM-IV diagnoses compared to the other instruments [44]). Knowing that this scale have limitation = not sure how this study is representative of population based prevalence of psychiatric disorder.

*There is no particular threat to the validity of the current study in light of having used the DAWBA as a diagnostic tool. The different time frame may, however, be useful to be aware of when comparing and contrasting prevalence estimates from different studies where other instruments and time frames may have been used. There is no reason to expect that the current estimates are more or less valid than previous estimates.*

Minor issues :

2nd paragraph (discussion gender and age difference): Anxiety disorders was higher for girls for anxiety disorders (anxiety disorder twice in a same sentence).

*Thank you for noticing this error. We have now corrected this sentence in the manuscript (page 9).*
